# Association of Temperament With Preoperative Anxiety in Pediatric Patients Undergoing Surgery: A Systematic Review and Meta-analysis

**DOI:** 10.1001/jamanetworkopen.2019.5614

**Published:** 2019-06-07

**Authors:** Cheryl H. T. Chow, Ayesha Rizwan, Richard Xu, Lauren Poulin, Varun Bhardwaj, Ryan J. Van Lieshout, Norman Buckley, Louis A. Schmidt

**Affiliations:** 1 Department of Psychology, Neuroscience, and Behaviour, McMaster University, Hamilton, Ontario, Canada; 2 Faculty of Medicine, University of Toronto, Toronto, Ontario, Canada; 3 Michael G. DeGroote School of Medicine, McMaster University, Hamilton, Ontario, Canada; 4 Clinical Psychology Graduate Program, York University, Toronto, Ontario, Canada; 5 Bachelor of Health Sciences Program, McMaster University, Hamilton, Ontario, Canada; 6 Department of Psychiatry and Behavioural Neurosciences, McMaster University, Hamilton, Ontario, Canada; 7 Department of Anesthesia, McMaster University, Hamilton, Ontario, Canada

## Abstract

**Importance:**

Preoperative anxiety is associated with poor behavioral adherence during anesthetic induction and adverse postoperative outcomes. Research suggests that temperament can affect preoperative anxiety and influence its short- and long-term effects, but these associations have not been systematically examined.

**Objective:**

To examine the associations of temperament with preoperative anxiety in young patients undergoing surgery.

**Data Sources:**

Studies from MEDLINE, Embase, CINAHL, PsycINFO, Web of Science, and the Cochrane Central Register of Controlled Trials databases were searched from database inception to June 2018.

**Study Selection:**

All prospective studies reporting associations of temperament with preoperative anxiety were included. Overall, 43 of 5451 identified studies met selection criteria.

**Data Extraction and Synthesis:**

Using the PRISMA guidelines, reviewers independently read 43 full-text articles, extracted data on eligible studies, and assessed the quality of each study. Data were pooled using the Lipsey and Wilson random-effects model.

**Main Outcomes and Measures:**

Primary outcome was the association of temperament with preoperative anxiety in patients undergoing surgery.

**Results:**

A total of 23 studies, with 4527 participants aged 1 to 18 years, were included in this review. Meta-analysis of 12 studies including 1064 participants revealed that emotionality (*r* = 0.11; 95% CI, 0.04 to 0.19), intensity of reaction (*r* = 0.29; 95% CI, 0.11 to 0.46), and withdrawal (*r* = 0.40; 95% CI, 0.23 to 0.55) were positively associated with preoperative anxiety, whereas activity level (*r* = −0.23; 95% CI, −0.31 to −0.16) was negatively correlated with preoperative anxiety. Impulsivity was not significantly associated with preoperative anxiety.

**Conclusions and Relevance:**

This systematic review and meta-analysis provided evidence suggesting that temperament may help identify pediatric patients at risk of preoperative anxiety and guide the design of prevention and intervention strategies. Future studies should continue to explore temperament and other factors influencing preoperative anxiety and their transactional effects to guide the development of precision treatment approaches and to optimize perioperative care.

## Key Points

**Question:**

Is temperament associated with preoperative anxiety in young patients undergoing surgery?

**Findings:**

In this systematic review of 23 studies including 4527 participants aged 1 to 18 years and meta-analysis of 12 studies including 1064 participants, certain temperament styles were associated with patients’ preoperative anxiety. Specifically, emotionality, intensity of reaction, and withdrawal were associated with increased preoperative anxiety, whereas activity level was associated with reduced anxiety.

**Meaning:**

Knowledge of temperamental propensity to preoperative anxiety in pediatric patients may help to guide the design of future detection, prevention, and/or individualized management strategies (eg, improving emotional regulation and coping skills) aimed at reducing the adverse effects of preoperative anxiety.

## Teaser

This systemic review and meta-analysis evaluates the associations of temperament with preoperative anxiety in pediatric patients aged 18 years and younger undergoing surgery.

## Introduction

Surgery can be a fearful event for many younger patients, as they face the threat of parental separation, loss of control, pain and discomfort, a strange environment, and uncertainty about the anesthetic procedure.^1,2^ The feelings of nervousness, worry, and tension related to an impending surgical experience have been formally recognized as preoperative anxiety,^1,3^ which can manifest as crying, anger, behavioral unrest, or verbal unrest.^1^

Nearly 5 million patients 18 years or younger in North America are at risk of developing preoperative anxiety each year.^4^ Preoperative anxiety is associated with important perioperative outcomes, including lengthened period of anesthetic induction and postoperative recovery.^1^ Higher levels of preoperative anxiety have also been associated with an increased risk of postoperative delirium, anxiety-related negative behavior changes, postoperative pain, and increased analgesia use.^5,6,7,8^ Given the adverse psychological and clinical implications of preoperative anxiety, identifying patients at greater risk presents a clinically important opportunity to improve their surgical experience and outcomes. Such knowledge can also help to inform a more appropriate allocation of finite hospital resources to patients who would most benefit from perioperative interventions.^9^

Previous research has found an association of temperament with anxiety in younger patients under stressful situations. Temperament is broadly defined as an individual’s characteristic nature or personality disposition, and it includes susceptibility to emotional stimulation, the strength and speed of response, the quality of the prevailing mood, the fluctuations and intensity of mood, and emotional regulation and reactivity.^10,11,12,13^ According to the diathesis-stress model, the interaction of individual vulnerability and stress leads to the development of psychopathology.^14^ Certain temperamental traits have been implicated as vulnerability factors for the development of psychological problems, such as anxiety and depression.^15,16^ Over the past 2 decades, behavioral inhibition, the tendency toward behavioral restraint and withdrawal in novel situations, has been widely studied and is thought to be an important risk factor for anxiety disorders.^17,18,19^ Meanwhile, negative affectivity or neuroticism, a temperamental sensitivity to negative stimuli, have also been implicated as a risk factor in the development of internalizing disorders, such as anxiety and depression.^15,20^ Thus, individual differences in temperament may provide important insights into which patients may fail to cope with or successfully manage challenging or novel situations (eg, surgery) and, as such, may be more likely to experience an elevated stress response.

Given the plethora of adverse outcomes associated with preoperative anxiety, the number of studies on temperament factors of preoperative anxiety has increased over the past 3 decades. However, these studies often appear in journals in disparate disciplines (eg, pediatrics, anesthesia, surgery, psychology) and yield conflicting results. For instance, some evidence has suggested that shy or inhibited patients may be at a greater risk of preoperative anxiety,^9^ whereas others have suggested that intensity of response, withdrawal, or low activity are risk factors.^21,22,23^ The growing extant literature on this topic coupled with a lack of consolidation or consensus among studies highlight the need for a systematic literature synthesis that provides an overview of the current state of knowledge of the association of temperament with preoperative anxiety.

To our knowledge, no systematic review has both qualitatively and quantitatively synthesized the available literature on associations of temperament with preoperative anxiety. Accordingly, we conducted a systematic review and meta-analysis of existing evidence to determine whether temperament is associated with preoperative anxiety in pediatric patients undergoing surgery under general anesthesia. These results could have important implications for the screening and identification of patients most at risk of preoperative anxiety while also helping to inform and guide the design of individualized prevention or intervention strategies.

## Methods

A protocol for this systematic review was registered on the PROSPERO international prospective register of systematic reviews (CRD42016038028).^24^ Both narrative and meta-analytic approaches (Preferred Reporting Items for Systematic Reviews and Meta-analyses [PRISMA] reporting guideline) were used to synthesize and analyze the data.^25^

### Selection Criteria

The research question for this systematic review was generated using the population, intervention (exposure), comparison, outcome, and study design approach. Study eligibility criteria as well as inclusion and exclusion criteria were also established using this framework. Prospective studies (ie, randomized clinical trials [RCTs], nonrandomized clinical trials, and observational study designs) that measured temperament before surgery were eligible for review.

The population of interest was patients aged 1 to 18 years undergoing surgery under general anesthesia at research, community, or university-affiliated hospitals. Only studies that measured temperament using validated scales (eg, Emotionality Activity Sociability Impulsivity [EASI] Temperament Scale) were eligible for inclusion. The outcome of interest was preoperative anxiety in patients undergoing surgery, as measured using validated anxiety scales (eg, the modified Yale Preoperative Anxiety Scale).

### Search Strategy

A search strategy was developed after consultation with a librarian at McMaster University. Systematic searches were conducted on articles published from database inception to June 2018 using 6 databases: MEDLINE, Embase, CINAHL, PsycINFO, Web of Science, and the Cochrane Central Register of Controlled Trials. No language restriction was applied. The search strategy used medical subject heading terms, which were combined with keywords if necessary (eAppendix in the Supplement). Reference lists were individually searched, and the results were included in this review.

### Study Screening

Two of us (C.H.T.C. and A.R.) independently screened titles and abstracts (κ = 75%). After screening, the review authors met and selected studies eligible for full-text screening. A third author (L.A.S.) was consulted to resolve disagreements.

### Data Extraction

A data extraction form was developed and piloted on 2 randomly selected studies included in the review. The information extracted from each study included study characteristics, population characteristics, details of the exposure, outcomes, summary of results, and risk of bias assessments. Risk of bias for RCTs and observational studies was assessed at the study level using the Cochrane Collaboration risk of bias tool and the Newcastle-Ottawa Scale, respectively.

### Statistical Analysis

A minimum of 2 studies was required for meta-analysis. The bivariate correlations (ie, Pearson correlation coefficients [*r*]) of child temperament with preoperative anxiety reported in available studies were used in meta-analyses. Using random-effects models of Lipsey and Wilson, we first converted all the correlation coefficients, *r*, for each study to a common metric using Fisher *z* transformations.^26,27^ The results were interpreted as significant if *r *did not cross the 0 line. We calculated the mean of *z*, inverse-variance weight, standard error of the mean of *z*, and *z* test for mean of *z*. We calculated 95% CIs using these formulas: lower = ES − 1.96(^se^_ES_) and upper = ES + 1.96(^se^_ES_), where ES indicates effect size and SE indicates standard error. Mean ESs were converted back to *r* for interpretation. Sensitivity analyses were performed on experimental studies for each temperament dimension to examine whether interventional effects were influencing the results. Cohen *d* criteria were used as a guideline for interpreting the size of mean ES: small, *r* = 0.10 to 0.29; medium, *r* = 0.30 to 0.49; and large, *r* = 0.50 or greater.^28^ Statistical analyses were conducted in Excel (Microsoft Corp).

## Results

### Study Characteristics

**Figure 1.**
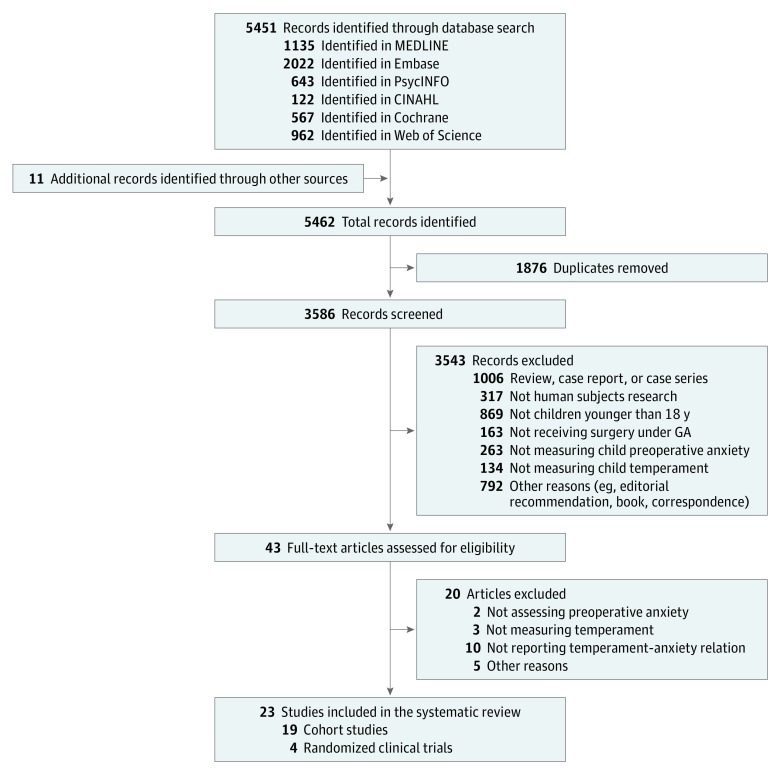
Flowchart of Study Selection Process Following PRISMA Guidelines GA indicates general anesthesia.

We identified 23 eligible studies (19 cohort studies and 4 RCTs) (Figure 1). A total of 4527 participants aged 1 to 18 years were included. Most studies were conducted in the United States (13 [57%]), followed by Canada (5 [22%]), Portugal (3 [13%]), Australia (1 [4%]), and the United Kingdom (1 [4%]).

### Risk of Bias

Within and across studies, all 4 RCTs demonstrated moderate to high risk of bias; 2 did not describe masking of participants and outcome in sufficient detail (eFigure in the Supplement). The overall Newcastle-Ottawa Scale scores on the 19 observational studies ranged from 5 to 7 (maximum score, 9). The use of self-report and lack of description in ascertainment of exposure were the most common sources of bias in the studies (eTable in the Supplement).

### Meta-analysis of Association of Temperament With Preoperative Anxiety

Data were available on 12 studies for meta-analysis and were pooled for 1064 unique participants.^5,21,23,29,30,31,32,33,34,35,36,37^ The included studies reported on the following temperamental traits: activity, emotionality, sociability, shyness, impulsivity, withdrawal, and intensity of reaction.

#### Activity

Activity is defined as the degree of energy expenditure through movement.^38^ The weighted average correlation from 7 studies^5,29,30,31,32,34,37^ (combined participants, 583) of the negative association of activity with preoperative anxiety was statistically significant with a small ES (*r* = −0.23; 95% CI, −0.31 to −0.16). Individual ESs ranged from −0.39 to −0.09. The negative correlation suggested that patients who scored as less active exhibited higher preoperative anxiety.

#### Emotionality

Emotionality is defined as the tendency to become easily and intensely upset.^39^ The weighted average correlation from 7 studies^23,30,31,32,33,35,37^ (combined participants, 680) of the association of emotionality with preoperative anxiety was statistically significant but had a small ES (*r* = 0.11; 95% CI, 0.04-0.19). Individual ESs ranged from −0.04 to 0.25. Overall, patients who scored higher on emotionality exhibited higher preoperative anxiety.

#### Sociability

Sociability is the tendency to seek social interactions.^40^ Among the 5 studies^30,31,34,36,37^ (combined participants, 338) that measured the negative association of sociability with preoperative anxiety, the weighted average correlation was −0.10 (95% CI, −0.21 to 0.01), a small ES. Individual ESs ranged from −0.37 to 0.09. These studies showed that patients who scored as less social exhibited higher preoperative anxiety.

#### Shyness

Shyness is defined as the tendency to avoid social interactions or situations.^41^ Among the 3 studies^30,31,32^ (combined participants, 285) that measured the association of shyness with preoperative anxiety, the weighted average correlation was 0.10 (95% CI, −0.02 to 0.22).^30,31,32^ Individual ESs ranged from 0.08 to 0.13. These studies indicated that patients who scored as more shy exhibited higher preoperative anxiety.

#### Impulsivity

Impulsivity is defined as the predisposition toward rapid, unplanned reactions to internal or external stimuli with diminished regard to the negative consequences of these reactions to the individual or to others.^42^ The weighted average correlation from 3 studies^23,33,37^ (combined participants, 133) of the association of impulsivity with preoperative anxiety was not significant (*r* = −0.01; 95% CI, −0.19 to 0.17). Individual ESs ranged from −0.26 to 0.44. This result suggested that higher impulsivity may not impart a greater risk of preoperative anxiety.

#### Withdrawal

Withdrawal is defined as the tendency to retreat from novel situations and people.^43^ The weighted average correlation from 2 studies^21,29^ (combined participants, 110) of the association of withdrawal with preoperative anxiety was statistically significant, with a medium ES (*r* = 0.40; 95% CI, 0.23-0.55). Individual ESs ranged from 0.29 to 0.60. These studies suggested that patients who were more withdrawn exhibited higher preoperative anxiety.

#### Intensity of Reaction

Intensity of reaction is defined as the typical strength of an individual’s responsiveness to a situation.^44^ The weighted average correlation from 2 studies^21,29^ (combined participants, 110) of the association of intensity of reaction with preoperative anxiety was statistically significant with a small ES (*r* = 0.29; 95% CI, 0.11-0.46). Individual ESs ranged from 0.27 to 0.33. These studies suggested that patients who had higher intensity of reaction exhibited higher preoperative anxiety.

### Sensitivity Analysis

**Figure 2.**
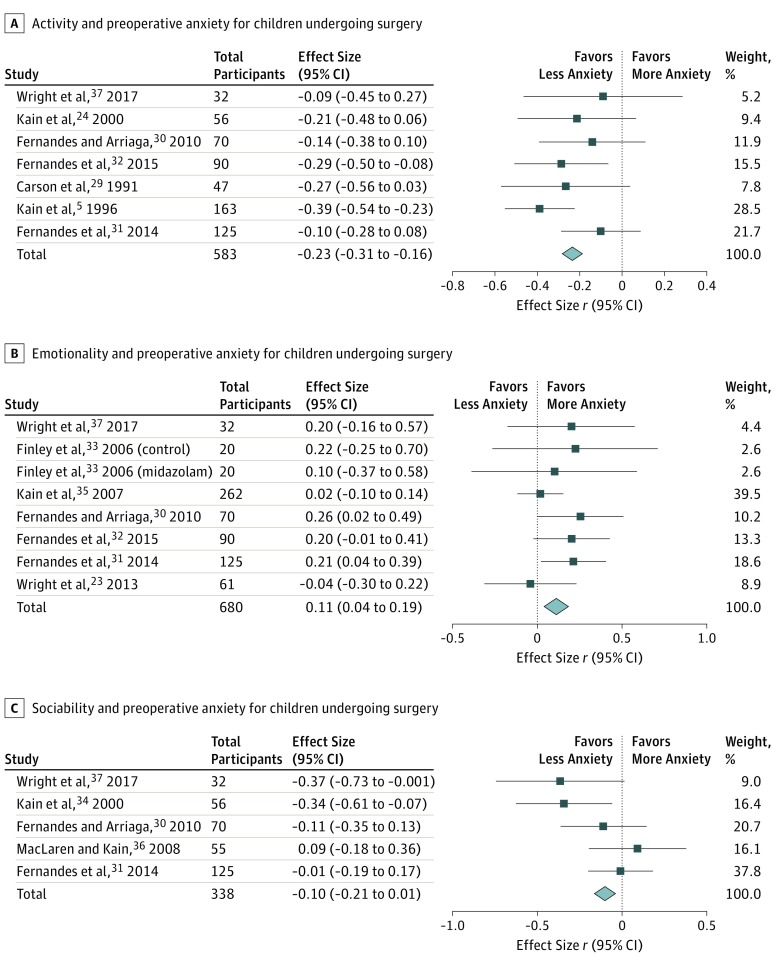
Meta-analyses of Association of Activity, Emotionality, and Sociability With Preoperative Anxiety

**Figure 3.**
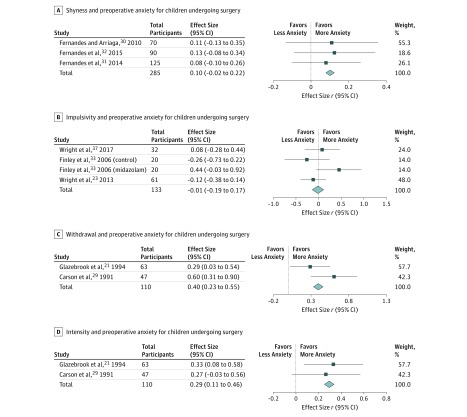
Meta-analyses of Association of Shyness, Impulsivity, Withdrawal, and Intensity of Reaction With Preoperative Anxiety

Sensitivity analyses were performed to assess whether experimental and observational studies generated different findings, and the results showed that the directionality of the weighted average correlation for certain temperament dimensions remained robust: sociability (number of studies, 2; *r* = −0.13; 95% CI, −0.31 to 0.06); impulsivity (number of studies, 2; *r* = −0.15; 95% CI, −0.36 to 0.08); and activity (number of studies, 3; *r* = −0.32; 95% CI, −0.42 to −0.21). Representative forest plots are shown in Figure 2 and Figure 3.

### Systematic Review

#### Activity

Overall, 3 studies^22,45,46^ reported the associations of activity with preoperative anxiety. Activity was found to be associated with higher anxiety in the preoperative holding area. A 1996 study^22^ found that activity also interacted with parental presence, and this interaction was associated with greater preoperative anxiety at anesthetic induction (Tukey test, 2.54; *P* = .01; *R^2^* = 0.15).

#### Emotionality

Of the 23 studies, only 1 study^45^ reported emotionality to be associated with greater preoperative anxiety. It found significant associations of emotionality with preoperative anxiety in the preoperative holding area and on separation at the operating room.

#### Sociability

Overall, 1 study reported on low sociability.^47^ It found low sociability to be associated with greater preoperative anxiety (β = −0.57; SE, 0.21; *P* = .007).

#### Shyness

Of the 23 studies, 2 studies^48,49^ reported the associations of shyness with preoperative anxiety. In Quinonez et al,^49^ shyness was significantly associated with anxiety during preseparation (*R*^2^ = 0.16; *F* = 9.23; *df* = 49; *P* = .003) and during separation from the parent at the entrance of the operating room (*R*^2^ = 0.10; *F* = 5.12; *df* = 49; *P* = .03). In a 2017 study,^48^ temperamental shyness was found to be associated with lower anxiety during the preoperative clinic visit (β = −10.78; *P* = .03) and in the holding area on the day of surgery (β = −12.31; *P* = .03).

#### Combined Temperamental Styles

In a 2004 study,^7^ patients at high risk, defined by exhibiting preoperative anxiety and postoperative maladaptive behavioral changes, were found to be more active, more emotional, and less sociable than patients in the low-risk group. A 2011 study^50^ also reported that greater preoperative anxiety was associated with internalizing behavior (*F*_1,47_ = 4.5; *P* = .04), somatic complaints (*F*_1,49_ = 4.0; *P* = .05), and fear (*F*_1,50_ = 5.2; *P* = .03). A 2006 study^51^ reported that patients with less anxiety scored lower on activity and impulsivity and that activity was associated with anxiety at anesthetic induction when the parent was present (*R*^2^ change = 0.016; *P* = .007).

In contrast, a 2006 study^46^ reported no difference in temperament styles between patients in the high-anxiety group vs patients in the low-anxiety group. Two other studies^52,53^ also reported nonsignificant associations of EASI temperamental dimensions with anxiety as well as easy vs difficult temperament with preoperative anxiety.

Study characteristics are summarized in Table 1. The scales used, the number of assessments and their time points, and outcome summaries for each study appear in Table 2.

**Table 1.** Selected Characteristics of 23 Studies in the Systematic Review

Source; Country	Study Design	Total Participants, No.	Age Range, y	Race/Ethnicity	Surgery Types	Intervention	Comparator Group
Carson et al,^29^ 1991; United States	Cohort	47	4-12	47 White patients (100%)	Tonsillectomy	NA	NA
Chow et al,^48^ 2017; Canada	Cohort	40	8-13	NS	Elective outpatient otolaryngologic surgery	NA	NA
Davidson et al,^53^ 2006; Australia	Cohort	1224	3-12	NS	Various procedures under general anesthesia	NA	NA
Fernandes and Arriaga,^30^ 2010; Portugal	Cohort (quasi-experimental design)	70	7-12	NS	Circumcision, herniorrhaphy, excision, orchiopexy, and cystoscopy	Clowns	No clowns
Fernandes et al,^31^ 2014; Portugal	RCT	125	8-12	NS	Minor and outpatient surgery, most commonly circumcision, excision, and herniorrhaphy	Educational material (booklet, video, or boardgame)	Entertainment material (booklet, video, or boardgame)
Fernandes et al,^32^ 2015; Portugal	RCT	90	8-12	NS	Minor and outpatient surgery, most commonly circumcision, excision, and herniorrhaphy	Educational multimedia application	Entertainment video game and control group
Finley et al,^33^ 2006; Canada	RCT	40	4-6	NS	Myringotomy with tympanostomy tube insertion	Midazolam (0.5 mg/kg) with acetaminophen (15 mg/kg)	Acetaminophen (15 mg/kg)
Fortier et al,^52^ 2009; United States	Cohort	143	7-17	92 White patients (64.3%), 9 Hispanic/Latino patients (6.3%), 9 African American patients (6.3%), 7 Asian American patients (4.9%), 7 multiracial patients (4.9%)	Various elective outpatient surgery	NA	NA
Fortier et al,^47^ 2010; United States	Cohort	261	2-12	210 White patients (80.5%), 27 African American patients (10.3%), 9 Hispanic/Latino patients (3.4%), 1 Asian American patient (0.4%), 15 patients with other race/ethnicity (5.7%)	Outpatient tonsillectomy and adenoidectomy	NA	NA
Fortier et al,^50^ 2011; United States	Cohort	59	11-18	NS	Various elective outpatient surgery	NA	NA
Glazebrook et al,^21^ 1994; United Kingdom	Cohort	63	2-11	NS	Nonspecified minor elective same-day surgery	NA	NA
Kain et al,^5^ 1996; United States	Cohort	163	2-10	NS	Elective ambulatory surgery (eg, myringotomy, herniorrhaphy)	NA	NA
Kain et al,^22^ 1996; United States	RCT	84	1-6	NS	Nonspecified elective outpatient surgery under general anesthesia	Parent present during anesthetic induction	Parent absent during anesthetic induction
Kain et al,^45^ 1996; United States	Cohort	143	2-10	NS	Elective outpatient surgery (eg, myringotomy, herniorrhaphy)	Behavioral preoperative preparation program: provide information, tour, role rehearsals (medical play) by child-life specialist	No behavioral preoperative preparation program
Kain et al,^34^ 2000; United States	Cohort	56	3-10	NS	Elective surgery, most commonly herniorrhaphy, tonsillectomy, and adenoidectomy	NA	NA
Kain et al,^7^ 2004; United States	Cohort	791	NS	NS	Nonspecified outpatient surgery under general anesthesia	NA	NA
Kain et al,^46^ 2006; United States	Cohort	241	5-12	202 White patients (83.8%)	Elective outpatient tonsillectomy and adenoidectomy	NA	NA
Kain et al,^51^ 2006; United States	Cohort	426	2-12	337 White patients (79.1%)	Nonspecified elective outpatient surgery under general anesthesia	NA	NA
Kain et al,^35^ 2007; United States	Cohort	262	2-10	209 White patients (79.8%), 19 African American patients (7.3%), 4 Hispanic patients (1.5%), 14 patients with other race/ethnicity (5.3%)	Elective surgery under general anesthesia	Midazolam (0.5 mg/kg, oral)	NA
MacLaren and Kain,^36^ 2008; United States	Cohort	55	6-12	48 White patients (87.2%)	Elective outpatient tonsillectomy and adenoidectomy	NA	NA
Quinonez et al,^49^ 1997; Canada	Cohort	51	2-5	21 White patients (41.2%), 20 First Nations patients (39.2%), 10 patients with other race/ethnicity (ie, African American and Hispanic) (19.6%)	Dental surgery	NA	NA
Wright et al,^23^ 2013; Canada	Cohort	61	3-6	55 White patients (90.1%)	Most ear, nose, and throat surgery (49 of 61 [80.3%])	NA	NA
Wright et al,^37^ 2017; Canada	Cohort	32	3-7	26 White patients (81.3%)	Same-day surgery (8 ear, nose, and throat [25.0%], 6 urology [18.8%], 5 dental [15.6%], 4 general [12.5%], 4 ophthalmologic [12.5%], 3 plastic [9.4%], 2 orthopedic [6.3%])	I-PPP	NA

Abbreviations: I-PPP, internet-delivered, preoperative preparation program; NA, not available or not applicable; NS, not specified; RCT, randomized clinical trial.

**Table 2.** Selected Outcome Summary of 23 Studies in the Systematic Review

Source; Country	Temperament Style	Temperament Scale	Temperament Assessment Time Point	Anxiety Scale	Anxiety Assessment Time Point	Outcome Summary
Carson et al,^29^ 1991; United States	Activity level, predictability, approach-withdrawal, adaptability to change, intensity of reaction, threshold of responsiveness, quality of mood, distractibility, and attention span/persistence; scores allowed for the creation of 4 subgroups: easy, difficult, slow to warm up, and intermediate (undifferentiated)	BSQ, MCTQ	7-10 d before surgery	PPRS	Soon after surgery	Higher predictability (*r* = −0.43), approach-withdrawal (*r* = −0.54), adaptability to change (*r* = −0.42), distractibility (*r* = 0.60), lower negative mood (*r* = −0.42), and lower response threshold (*r* = 0.50) were significantly associated with less distress during hospital procedures and better in-hospital adjustment; intensity of reaction was positively associated with in-hospital adjustment (*F* = 11.48; *R*^2^ = .14; *P* < .001; β = 0.42; *t* = 3.14; *P* < .01)
Chow et al,^48^ 2017; Canada	Shyness, sociability, emotionality, activity, attention span/persistence, and soothability	CCTI	Preoperative clinic visit approximately 1 wk before surgery	CPMAS	Preoperative clinic visit approximately 1 wk before surgery and same-day surgery holding area immediately before surgery	Temperamental shyness was associated with lower preoperative anxiety at clinical visit (β = −10.78; *P* = .03) and in same-day surgery holding area (β = −12.31; *P* = .03)
Davidson et al,^53^ 2006; Australia	Easy or difficult temperament	STST, SATI	Day of surgery	mYPAS	Immediately before anesthetic induction	No association of temperament style with anxiety
Fernandes and Arriaga,^30^ 2010; Portugal	Emotionality, activity, sociability, and shyness	EAS-TS	Day of surgery	CSWQ, SAM	Preoperative holding area (CSWQ, SAM) and postoperative (SAM)	Higher emotionality was significantly associated with greater worries about surgery (*r* = 0.25); more sociable patients were significantly associated with higher preoperative positive affect (*r* = 0.29)
Fernandes et al,^31^ 2014; Portugal	Emotionality, activity, sociability, and shyness	EAS-P	Day of surgery	CSWQ (global preoperative worries only), SAM	Preintervention (SAM), postintervention (SAM, CSWQ), after surgery (SAM)	Emotionality was significantly associated with global worries (*r* = 0.21); other temperament dimensions were not significantly associated with preoperative worry: shyness (*r* = 0.08), activity (*r* = −0.10), and sociability (*r* = −0.01)
Fernandes et al,^32^ 2015; Portugal	Emotionality, activity, sociability, and shyness	EAS-P	Day of surgery	CSWQ, SAM	Preintervention (SAM), postintervention (CSWQ, SAM)	Lower activity was significantly associated with global worries (*r* = −0.28); increased emotionality, lower shyness, and lower activity were significantly associated with higher hospitalization worries (emotionality: *r* = 0.25; shyness: *r* = −0.24; activity: *r* = −0.44); higher emotionality and lower activity were significantly associated with greater illness worries (emotionality: *r* = 0.27; activity: *r* = −0.21); activity levels were significantly associated with worries about surgery (β = −0.231; *t* = −3.042; *P* = .003)
Finley et al,^33^ 2006; Canada	Emotionality, activity, sociability, and impulsivity	EASI	1 wk before surgery	mYPAS without parent rating	Predrug administration (90 min before surgery), 20 min after drug administration (40 min before surgery), and at anesthetic induction	In midazolam group, there was a significant positive association of anxiety at anesthetic induction with the impulsivity dimension (*r* = 0.42)
Fortier et al,^52^ 2009; United States	Emotionality, activity, sociability, impulsivity, and avoidant temperament	EASI, CDI (avoidant temperament)	Day of surgery	STAI-CH	Preoperative holding area	Association of EASI with anxiety not investigated; association of avoidance with anxiety was not significant; bivariate correlations showed that state anxiety was negatively correlated with avoidance (*r* = −0.22; *P* = .02); however, analysis of variance of patients who were highly avoidant (ie, upper 25%) and not avoidant (ie, lower 25%) showed that mean differences for state anxiety between the 2 groups was not significant (*F*_1,116_ = 2.96; *P* = .08)
Fortier et al,^47^ 2010; United States	Emotionality, activity, sociability, impulsivity, internalizing problems, and externalizing problems	EASI, CBCL	7-10 d before surgery	mYPAS, VAS, NRS	Preoperative holding area (mYPAS, VAS); parental separation (mYPAS, VAS); OR entrance (mYPAS, VAS); introduction to anesthesia mask (mYPAS, VAS); arrival to recovery (VAS); 6 h, 12 h, 18 h, and 24 h in recovery (VAS); 2 d, 3 d, 7 d, and 14 d after operation (NRS)	Low child sociability was significantly associated with perioperative anxiety (β = −0.57; SE, 0.21; odds ratio, 0.56; 95% CI, 0.37-0.85; *P* = .007); patients in the high-anxiety group (1 SD above mean perioperative anxiety score) were associated with lower sociability than patients in the low-anxiety group (high-anxiety group mean [SD] EASI sociability score, 18.1 [2.7]; low-anxiety group mean [SD] EASI sociability score, 19.6 [2.1]; *P* = .03)
Fortier et al,^50^ 2011; United States	Activation/control, affiliation, attention, fear, frustration, inhibitory control, shyness, aggression, depressive mood, surgency, anxious/depressed, withdrawn/depressed, somatic complaints, social problems, thought problems, attention problems, rule-breaking behavior, aggressive behavior, internalizing problems, and externalizing problems	EATQ-R-P, CBCL/6-18	In preoperative holding area or OR prior to surgery	VAS, ECG, SCL, BP	Preoperative holding area (VAS, BP, ECG, SCL), parental separation (VAS), introduction to anesthesia mask (VAS, BP, ECG, SCL)	High preoperative anxiety assessed by the VAS was associated with internalizing behavior (*F*_1,47_ = 4.5; *P* = .04), somatic complaints (*F*_1,49_ = 4.0; *P* = .05), and fear (*F*_1,50_ = 5.2; *P* = .03)
Glazebrook et al,^21^ 1994; England	Activity level, predictability, approach-withdrawal, adaptability to change, intensity of reaction, threshold of responsiveness, quality of mood, distractibility, attention span/persistence, and normal vs extreme behavior	TS, BQ (normal vs extreme behavior)	Day before surgery	OSBD	OR	OSBD distress associated with extreme behavior (*r* = 0.88), intensity of reaction (*r* = 0.32), withdrawal (*r* = 0.28); intensity of reaction was associated with distress (*R*^2^ for model = 0.21; *F*_2,47_ = 6.2); patients in the high-distress group (scoring 4 or greater on OSBD distress scale) scored significantly higher on intensity of reaction (*z* = 2.47) and withdrawal (*z* = 2.22)
Kain et al,^5^ 1996; United States	Emotionality, activity, sociability, and impulsivity	EASI	Day of surgery	CARS, VAS, VPT	Preoperative holding area (VPT, VAS), parental separation (VAS, CARS)	Low activity scores were significantly associated with high child anxiety (*r* = −0.37) in preoperative waiting room (*R*^2^ = 0.11; *F* = 6.1) and at separation to the OR (*R*^2^ = 0.18; *F* = 12.3)
Kain et al,^22^ 1996; United States	Emotionality, activity, sociability, and impulsivity	EASI	1 wk before surgery	VAS, YPAS, CARS, cortisol analysis	Preoperative holding area (VAS), OR entrance (YPAS, CARS), introduction of anesthesia mask (YPAS, CARS), after induction (cortisol analysis)	Activity dimension interacted with parental presence and was associated with child anxiety, as measured by serum cortisol (*t* = 2.54; *P* = .01; *R*^2^ = 0.15)
Kain et al,^45^ 1996; United States	Emotionality, activity, sociability, and impulsivity	EASI	1-2 d before surgery	VAS, VPT, CARS	Preoperative holding area (VAS, VPT), parental separation (VAS, CARS)	Activity was significantly associated with child anxiety in preoperative holding area; emotionally labile patients (upper quartile of EASI emotionality scale) who received a behavioral preparation program were associated with more anxiety than emotionally stable patients (lower quartile of EASI emotionality scale) in preoperative holding area (upper-quartile group mean [SD] VAS score, 51 [16]; lower-quartile group mean [SD] VAS score, 34 [19]; *P* = .03) and on separation to the OR (upper-quartile group mean [SD] VAS score, 41 [19]; lower-quartile group mean [SD] VAS score, 13 [8]; *P* = .01)
Kain et al,^34^ 2000; United States	Emotionality, activity, sociability, and impulsivity	EASI	1 wk prior to surgery	mYPAS	Preoperative holding area, OR entrance, introduction of anesthesia mask	Lower activity (*r* = −0.21) and lower sociability (*r* = −0.33) were significantly associated with high perioperative anxiety; child sociability was an independent risk factor for perioperative anxiety (*R*^2^ = 0.38; *F* = 5.5; *P* = .04)
Kain et al,^7^ 2004; United States	Emotionality, activity, sociability, and impulsivity	EASI	NS	mYPAS	Preoperative holding area, OR entrance, introduction of anesthesia mask	Compared with children at low risk, children at high risk (those with emergence delirium and intense preoperative anxiety or postoperative maladaptive behaviors) were associated with higher emotionality (high-risk group mean [SD] EASI emotionality score, 11.5 [3.4]; low-risk group mean [SD] EASI emotionality score, 10.2 [3.6]; *P =* .03), more activity (high-risk group mean [SD] EASI activity score, 17.0 [4.5]; low-risk group mean [SD] EASI activity score, 15.2 [4.2]; *P* = .02), and less sociability (high-risk group mean [SD] EASI sociability score, 17.7 [3.4]; low-risk group mean [SD] EASI socialiability score, 18.8 [2.8]; *P* = .01)
Kain et al,^46^ 2006; United States	Emotionality, activity, sociability, and impulsivity	EASI	7-10 d before surgery	mYPAS	Preoperative holding area, introduction of anesthesia mask	No difference in temperament styles between the high-anxiety and low-anxiety groups
Kain et al,^51^ 2006; United States	Emotionality, activity, sociability, and impulsivity	EASI	Day of surgery	mYPAS	Preoperative holding area, introduction of anesthesia mask	Compared with the low-anxiety group, the high-anxiety group (upper quartile of mYPAS score during induction) was associated with higher activity (high-anxiety group mean [SD] EASI activity score, 17.8 [3.9]; low-anxiety group mean [SD] EASI activity score, 15.6 [3.9]; *P* = .001) and impulsivity levels (high-anxiety group mean [SD] EASI impulsivity score, 13.9 [3.6]; low-anxiety group mean [SD] EASI impulsivity score, 12.5 [3.7]; *P* = .005); activity dimension was associated with anxiety at anesthetic induction while parent was present (*R*^2^ change = 0.016; *P* = .007)
Kain et al,^35^ 2007; United States	Emotionality, activity, sociability, and impulsivity	EASI	Day before surgery	mYPAS	Preoperative holding area, separation to the OR, OR entrance, introduction of anesthesia mask	EASI emotionality was significantly associated with child anxiety at induction (ρ = 0.174); logistic regression model found emotionality to be a significant predictor of preoperative anxiety (β = 0.209; SE, 0.06; Wald χ^2^ = 11.89; *P* = .001); patients who did not respond to midazolam scored higher on the EASI emotionality subscale than patients who did respond (nonresponder mean [SD] EASI emotionality score, 13.6 [3.6]; responder mean [SD] EASI emotionality score, 11.3 [3.8]; *P* = .001)
MacLaren and Kain,^36^ 2008; United States	Emotionality, activity, and sociability	EASI	5-7 d Before surgery	mYPAS	Preoperative holding area, OR entrance	No correlation was found between child anxiety at induction and sociability (*r* = 0.09); association of other temperament dimensions and anxiety was not assessed
Quinonez et al,^49^ 1997; Canada	Emotionality, activity, sociability, and shyness	EAS-TS	Prior to OR on day of surgery	MBPRS-R, Frankl Scale	Prior to OR (ie, in playroom), en route to OR, parental separation, postseparation/anesthetic induction	Shyness was significantly associated with state anxiety (disruptive behavior, as measured by MBPRS-R) during preseparation (*R*^2^ = 0.16; *F* = 9.23; *P* = .004) and separation of the child from the parent at the entry of the OR (*R*^2^ = 0.10; *F* = 5.12; *P* = .03)
Wight et al,^23^ 2013; Canada	Emotionality, activity, sociability, impulsivity; anxiety-shyness, and hyperactivity-impulsivity	EASI; CPRS-R: L	Prior to day of surgery	mYPAS	Introduction of anesthesia mask	CPRS-R:L anxiety-shyness subscale was significantly associated with preoperative anxiety at anesthetic induction (*r* = 0.24)
Wright et al,^37^ 2017; Canada	Emotionality, activity, sociability, and impulsivity	EASI	Prior to day of surgery	mYPAS	Admission, day surgery unit, OR entrance, introduction of anesthesia mask	No significant association of temperament dimensions and child preoperative anxiety

Abbreviations: BP, blood pressure; BQ, Scaife and Campbell behavioral questionnaire; BSQ, Behavioral Style Questionnaire; CARS, Clinical Anxiety Rating Scale; CBCL, Child Behavior Checklist; CBCL/6-18, Child Behavior Checklist for Ages 6-18; CCTI, Colorado Child Temperament Inventory; CDI, children’s desire for information; CPMAS, Children’s Perioperative Multidimensional Anxiety Scale; CPRS-R:L, Conners Parent Rating Scale–Revised, long form; CSWQ, Child Surgery Worries Questionnaire; EASI, Emotionality Activity Sociability and Impulsivity inventory; EAS-P and EAS-TS, Emotionality Activity Sociability temperament survey; EATQ-R-P, Early Adolescent Temperament Questionnaire Revised–parent version; ECG, electrocardiography; MBPRS-R, Melamed Behavior Profile Rating Scale–Revised; MCTQ, Middle Childhood Temperament Questionnaire; mYPAS, modified Yale Preoperative Anxiety Scale; NRS, numeric rating scale; NS, not specified; OR, operating room; OSBD, Observational Scale Of Behavioral Distress; PPRS, Pediatric Patient Rating Scale; SAM, self-assessment mannequin; SATI, School Age Temperament Inventory; SCL, skin conductance level; STAI-CH, State Trait Anxiety Inventory for Children; STST, Short Temperament Scale for Toddlers; TS, temperament scale; VAS, visual analog scale; VPT, Venham Picture test; YPAS, Yale Preoperative Anxiety Scale.

## Discussion

To our knowledge, this is the first systematic review and meta-analysis to examine the association of temperament with preoperative anxiety among pediatric patients. This review provided evidence that individual differences in temperament may help identify young patients at risk of preoperative anxiety and guide the design of future prevention and intervention strategies. This review included 23 studies (observational and experimental), involving 4527 participants aged 1 to 18 years undergoing elective same-day surgery. The meta-analytic results of 12 pooled studies revealed that certain temperament styles were significantly associated with preoperative anxiety. Specifically, emotionality, intensity of reaction, and withdrawal were found to be associated with increased preoperative anxiety, whereas activity level was associated with less anxiety. The ESs ranged from small to medium. Impulsivity was not associated with preoperative anxiety.

Our findings are consistent with previous research investigating the association of temperament with psychopathology in children and youth in other stressful, nonclinical contexts^54,55^ and extends this work by examining these associations in the surgical setting. Importantly, this broadens our understanding of the development of anxiety in a distinct clinical context. Our results suggest that both negative emotionality (small ES) and high intensity of reaction (small ES) were associated with preoperative anxiety. These findings are congruent with previous longitudinal studies, in which negative emotionality in infancy and middle childhood were found to be associated with anxious behaviors 2 years later^56^ as well as with anxiety symptoms in adulthood.^57,58^ The results of this meta-analysis are also consistent with a 2007 study,^59^ which found that negative emotionality was associated with dental fear, as were shyness and activity.

We also noted the association of inhibited temperament (ie, shyness, withdrawal behaviors) with preoperative anxiety, consistent with previous literature examining this association in everyday normative contexts, such as school and home.^60,61,62,63,64^ Our findings also suggested a negative association of sociability with preoperative anxiety. We found that shyness and sociability exerted small ESs, while withdrawal behaviors exerted medium ESs. The modest ES magnitudes can be understood in the context of research indicating that stronger effects are only seen when inhibited temperament in early childhood is combined with other risk factors, such as parental factors or psychophysiological reactivity.^65^ Thus, temperamental traits should be examined within a biopsychosocial framework, which includes both biological factors (ie, age, sex, or physiological reactivity) and environmental moderating factors (ie, socioeconomic status, previous surgical experiences, or parental anxiety) to best predict preoperative anxiety and guide future directions for tailored, individualized approaches to managing preoperative anxiety.

In terms of activity level, our results showed that low activity (small ES) was associated with higher preoperative anxiety in patients undergoing surgery. This result is consistent with a longitudinal study^58^ that showed negative associations of activity levels with anxious behaviors at ages 4 years and 8 to 9 years. Finally, the association of impulsivity with preoperative anxiety in patients undergoing surgery was not significant. This might be explained by the fact that impulsivity is commonly associated with externalizing behaviors, such as aggression, delinquency, and hyperactivity, but not internalizing problems, such as anxiety.^66^ This is further supported by studies that showed patients with externalizing problems were more impulsive than patients with internalizing problems.^67,68,69^

Taken together, patients with negative emotionality and/or inhibited temperaments seem more prone to experiencing preoperative anxiety. Patients with behavioral inhibition or a so-called difficult temperament (eg, negative mood, slow to adapt to new situations) are reported to be at a heightened risk of developing anxiety disorders later in life. Particularly, a difficult temperament was identified as the single most important risk factor for heightened anxiety symptoms.^70^ This can be further explained by the view that inhibited temperament reflects low temperamental behavioral reactivity.^69^ Patients who are behaviorally inhibited appear to be more rigid and inflexible in novel or stressful contexts,^68^ and this inability to adapt may predispose them to greater anxiety in an unfamiliar and stress-inducing environment, like the surgical setting.

### Strengths and Implications

The present review has a number of important clinical implications. Its findings contribute to the body of evidence supporting the relevance of temperament in the development and/or maintenance of anxiety. This review provides support that certain temperamental traits (ie, emotionality and withdrawal) might be risk factors for preoperative anxiety and may predict how patients will respond in this unique and stressful setting. This knowledge can be used to help with refinement of screening processes and prevention strategies for preoperative anxiety and to design interventions (eg, improving emotional regulation and coping skills). As temperament represents only a single risk factor, future research should continue to study these individual-level characteristics with other individual-level (eg, psychophysiological reactivity) and family-level (eg, parental behaviors) factors to develop more holistic prognostic models with greater predictive potential.

### Limitations

Several limitations should be noted. First, none of the reviewed studies examined the associations of temperament with postoperative outcomes (eg, pain, emergence delirium, recovery) beyond anxiety. Second, quantitative correlational data for the meta-analysis were only available for a limited number of studies (12), as some of the studies reviewed did not report usable ES statistics for temperament and anxiety. However, the narrative summary from these studies generally showed congruent results. Third, only 2 or 3 studies examined certain temperament dimensions. Fourth, study designs were variable (ie, observational vs experimental). However, sensitivity analyses revealed that most results remained robust when these variations were accounted for, with some demonstrating even stronger effects, whereas others were attenuated owing to a lack of statistical power. Fifth, the included studies analyzed only a subset of the various temperament dimensions that have been implicated in anxiety and/or internalizing disorders. Other temperament traits that are associated with psychopathology, such as surgency, should be considered in future work.^67^ Sixth, subjective reports on temperament and/or anxiety (eg, by parents, patients, or research staff) are prone to reporting bias and interobserver bias. Seventh, the standards for the collection of temperament and anxiety data (eg, measurement or timing) have not been established and varied across studies. In this review, the EASI Temperament Scale was the primary temperament measure in 18 of 23 studies. Although the EASI Temperament Scale has been widely used as a measure of temperament in the literature, a 2017 systematic review conducted by Walker et al^71^ suggested that the EASI Temperament Scale may have inconsistent psychometric properties with variable internal consistency and poor factor structure. Thus, future studies should use different, more psychometrically sound measures of temperament and/or include a modified version of the EASI Temperament Scale to improve the reliability and validity of results. Future studies should also take into consideration the timing of temperament measures, as the concurrent assessments of temperament and preoperative anxiety might result in inflated estimates of ESs associating temperament with anxiety. Although the findings of this review are informative, future studies should address the aforementioned limitations in design and data collection to provide more definitive and robust conclusions that can guide future clinical practice.

## Conclusions

Our systematic review and meta-analysis suggests that temperament styles are significantly associated with preoperative anxiety for young patients undergoing surgery. The findings showed that patients with negative emotionality and inhibited temperament are more prone to experiencing preoperative anxiety, whereas active and social patients are less likely to experience preoperative anxiety. Future studies should continue delineating the role of temperament with other biological and environmental determinants of preoperative anxiety and their transactional effects by using more standardized measures, such as behavioral observations or noninvasive physiological measures (eg, cortisol or electrocortical activity). Furthermore, future studies should examine the association of temperament with other perioperative outcomes that are of significance to patients, families, and practitioners, such as postoperative pain, emergence delirium, and postoperative maladaptive behaviors, to advance precision medicine approaches in perioperative management. Given the negative impact of preoperative anxiety, identifying etiological factors that may predict its emergence can help to guide the design of future detection, prevention, and individualized management strategies aimed at reducing the adverse effects of preoperative anxiety.

## Notes

Supplement. **eAppendix.** MEDLINE Search Strategy **eFigure.** Risk of Bias Assessment for Randomized Clinical Trials **eTable.** Risk of Bias Assessment for Observational Studies
