# Supplementary material for: Association of Temperament With Preoperative Anxiety in Pediatric Patients Undergoing Surgery: A Systematic Review and Meta-analysis
Source: JAMA Netw Open. 2019 Jun 7;2(6):e195614. doi: 10.1001/jamanetworkopen.2019.5614 (PMC6563582; doi:10.1001/jamanetworkopen.2019.5614)
Supplement: Supplement. — eAppendix. MEDLINE Search Strategy eFigure. Risk of Bias Assessment for Randomized Clinical Trials eTable. Risk of Bias Assessment for Observational Studies [file jamanetwopen-2-e195614-s001.pdf]

Supplementary Online Content

Chow CHT, Rizwan A, Xu R, et al. Association of temperament with preoperative anxiety in pediatric patients undergoing surgery: a systematic review and meta-analysis. *JAMA Netw Open*. 2019;2(6):e195614. doi:10.1001/jamanetworkopen.2019.5614

eAppendix. MEDLINE Search Strategy

eFigure. Risk of Bias Assessment for Randomized Clinical Trials

eTable. Risk of Bias Assessment for Observational Studies

This supplementary material has been provided by the authors to give readers additional information about their work.

© 2019 Chow CHT et al. *JAMA Network Open.*

eAppendix: MEDLINE Search Strategy

1. Anxiety/
2. anxiety.mp.
3. anxiet*.mp.
4. anxious.mp.
5. nervousness.mp.
6. Fear/
7. fear.mp.
8. Panic/
9. panicked.mp.
10. distress.mp.
11. emotional stress.mp.
12. Stress, Psychological/
13. psychological stress.mp.
14. feel* of apprehension.mp.
15. feel* of dread.mp.
16. feel* of worry.mp.
17. feel* of worried.mp.
18. feel* of terror.mp.
19. 1 or 2 or 3 or 4 or 5 or 6 or 7 or 8 or 9 or 10 or 11 or 12 or 13 or 14 or 15 or 16 or 17 or 18
20. Preoperative Care/
21. Preoperative Period/
22. preoperative.mp.
23. surgical procedures.mp.
24. Surgical Procedures, Operative/
25. operative.mp.
26. surgery.mp.
27. surgical.mp.
28. preop*.mp.
29. pre-op*.mp.
30. pre-op.mp.
31. periop*.mp.
32. peri-op*.mp.
33. preoperat*.mp.
34. Temperament/
35. temperament.mp.
36. Personality/
37. personality.mp.
38. shyness/
39. shyness.mp.
40. sociability.mp.
41. emotionality.mp.
42. impulsivity.mp.
43. "Inhibition (Psychology)"/

© 2019 Chow CHT et al. *JAMA Network Open.*

44. inhibition.mp.
45. soothability.mp.
46. attention span-persistence.mp.
47. 34 or 35 or 36 or 37 or 38 or 39 or 40 or 41 or 42 or 43 or 44 or 45 or 46
48. 20 or 21 or 22 or 23 or 24 or 25 or 26 or 27 or 28 or 29 or 30 or 31 or 32 or 33
49. 19 and 47 and 48

© 2019 Chow CHT et al. *JAMA Network Open.*

eFigure. Risk of Bias Assessment for Randomized Clinical Trials

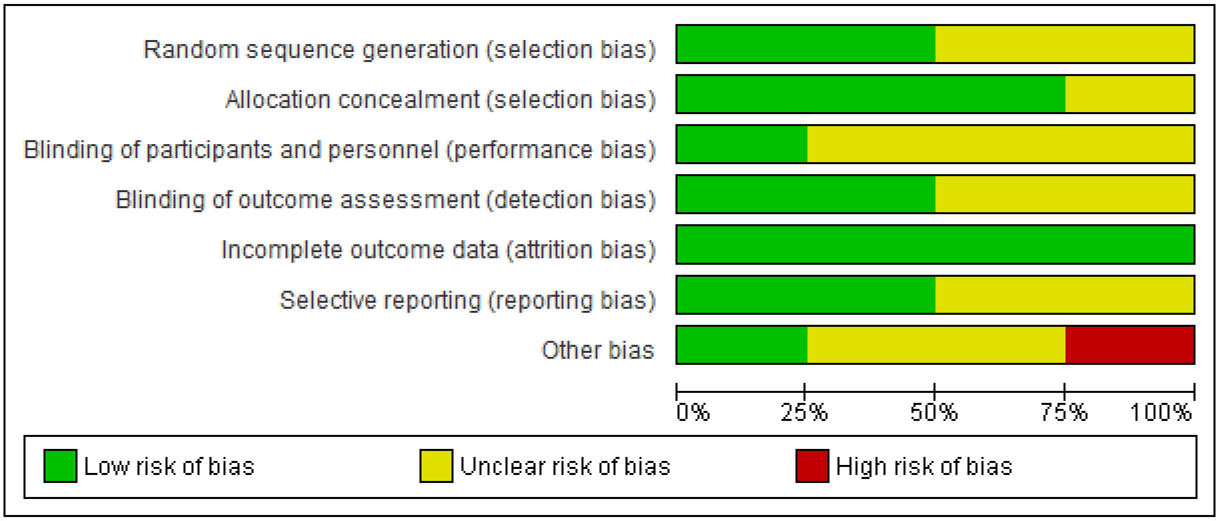

© 2019 Chow CHT et al. *JAMA Network Open.*

eTable. Risk of Bias Assessment for Observational Studies

**Study authors**	**Year, country**	**Selection**	**Comparability**	**Outcome**	**Total Score**
Carson et al.	1991, USA	2	2	1	5
Chow et al.	2017, Canada	2	1	2	5
Davidson et al.	2006, Australia	2	1	2	5
Fernandes& Arriaga	2010, Portugal	3	2	1	6
Fortier et al.	2009, USA	2	1	2	5
Fortier et al.	2010, USA	3	2	2	7
Fortier et al.	2011, USA	2	2	2	6
Glazebrook et al.	1994, England	2	0	3	5
Kain et al.	1996a, USA	2	2	1	5
Kain et al.	1996c, USA	2	1	2	5
Kain et al.	2000, USA	2	2	2	6
Kain et al.	2004, USA	2	2	2	6
Kain et al.	2006a, USA	2	1	2	5
Kain et al.	2006b, USA	2	1	2	5

© 2019 Chow CHT et al. *JAMA Network Open.*

Kain et al.	2007, USA	2	2	2	6
MacLaren&Kain	2007, USA	2	1	3	6
Quinonez et al.	1997, Canada	2	2	2	6
Wright et al.	2013, Canada	2	2	3	7
Wright et al.	2017, Canada	2	2	2	6

© 2019 Chow CHT et al. *JAMA Network Open.*
